# Supplementary material for: A quantitative description of the transition between intuitive altruism and rational deliberation in iterated Prisoner’s Dilemma experiments
Source: Sci Rep. 2019 Nov 19;9:17046. doi: 10.1038/s41598-019-52359-3 (PMC6864093; doi:10.1038/s41598-019-52359-3)
Supplement: Supplementary file 1 — Supplementary Information [file 41598_2019_52359_MOESM1_ESM.pdf]

Supplementary Information for:
A quantitative description of the transition between intuitive altruism and rational deliberation in iterated Prisoner's Dilemma experiments

Riccardo Gallotti[1,2,3,*] **and Jelena Grujić**[4,5]

[1]Instituto de Física Interdisciplinar y Sistemas Complejos IFISC (CSIC-UIB), Palma de Mallorca, Spain.
[2]Center for Complex Systems & Brain Sciences (CEMSC[3]), Universidad Nacional de San Martin, Buenos Aires, Argentina.
[3]Fondazione Bruno Kessler, Trento, Italy.
[4]AI lab, Computer Science Department, Vrije Universiteit Brussel, Brussels, Belgium.
[5]MLG, Département d'Informatique, Université Libre de Bruxelles, Brussels, Belgium.
[*]To whom correspondence should be addressed; E-mail: rgallotti@gmail.com.

Supplementary Figures and Tables

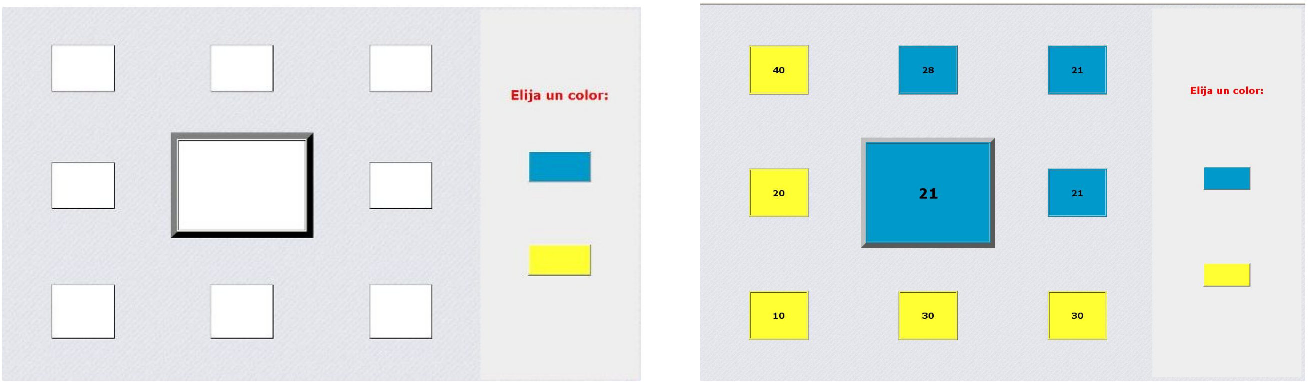

Supplementary Figure 1. The interface of the multiplayer experiment. On the left: the screen in the first round of each phase. On the right: the screen in the following rounds.

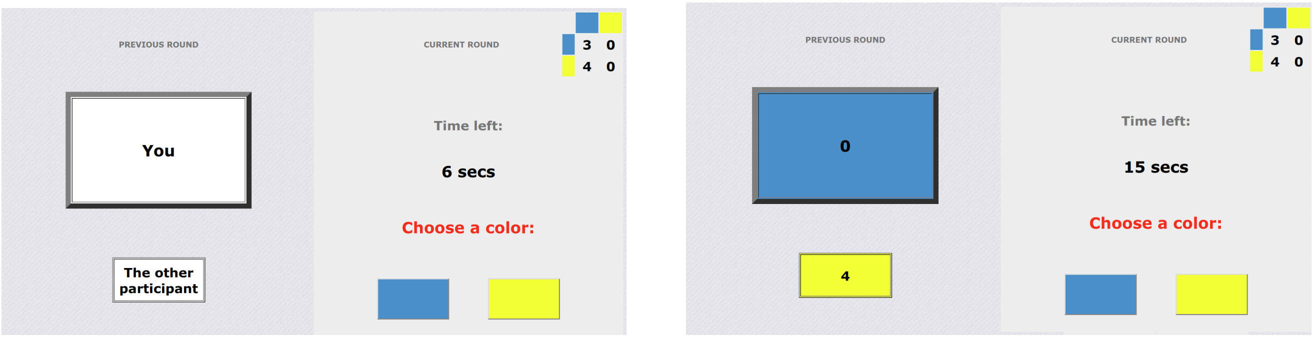

Supplementary Figure 2. The interface of the pairwise experiment. On the left: the screen in the first round of each phase. On the right: the screen in the following rounds.

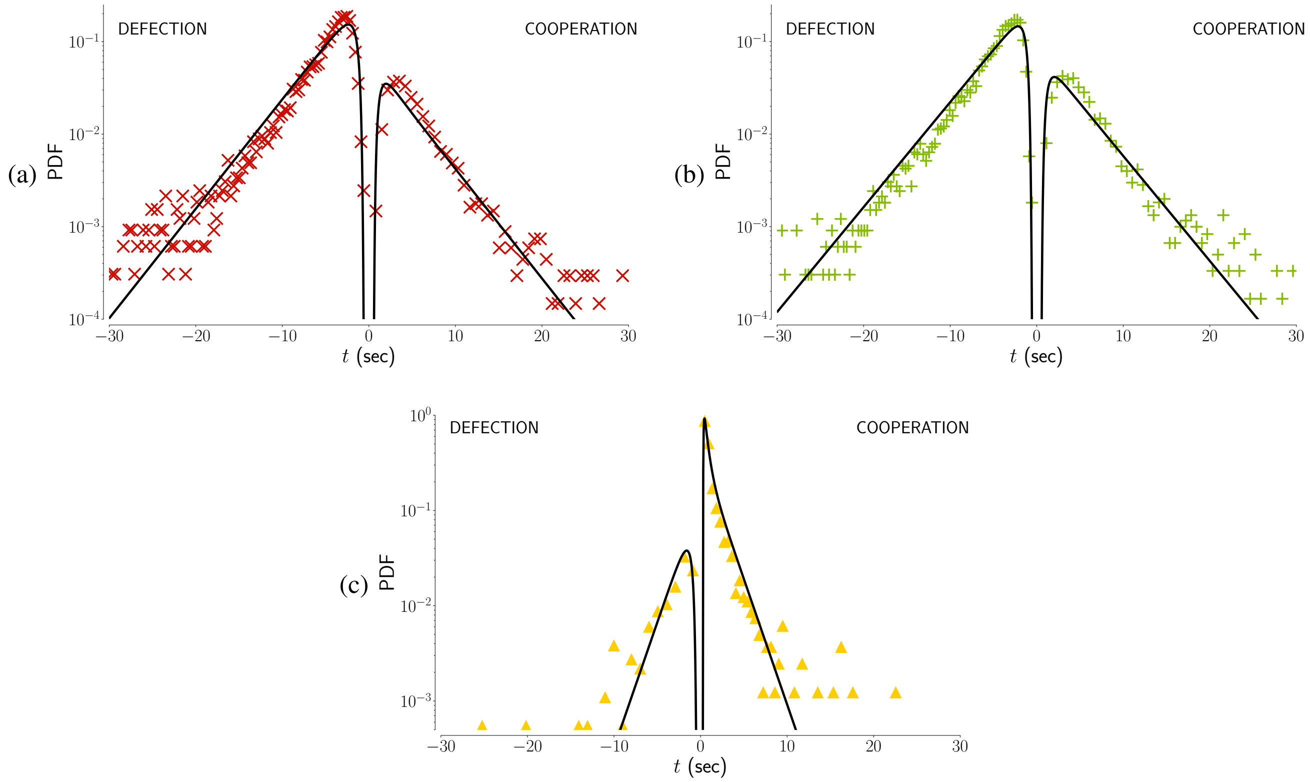

Supplementary Figure 3. Experimental distribution fitted with the theoretical curves for the DDM. (a) Multiplayer experiment, random phase ($r^2 = 0.94$). **(b)** Multiplayer experiment, fix 2 phase ($r^2 = 0.95$). **(c)** Pairwise experiment ($r^2 = 0.99$).

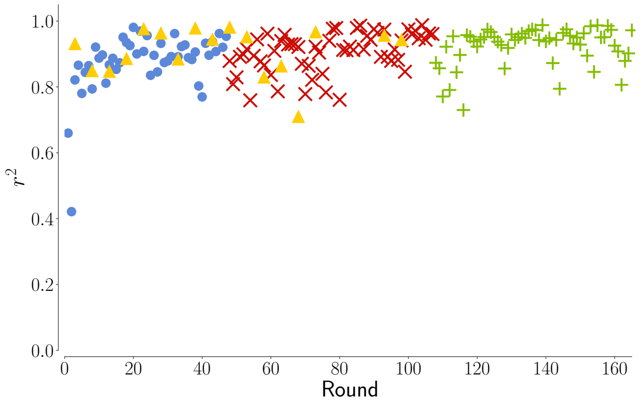

Supplementary Figure 4. r^2 value of the fits described in Figure 3.

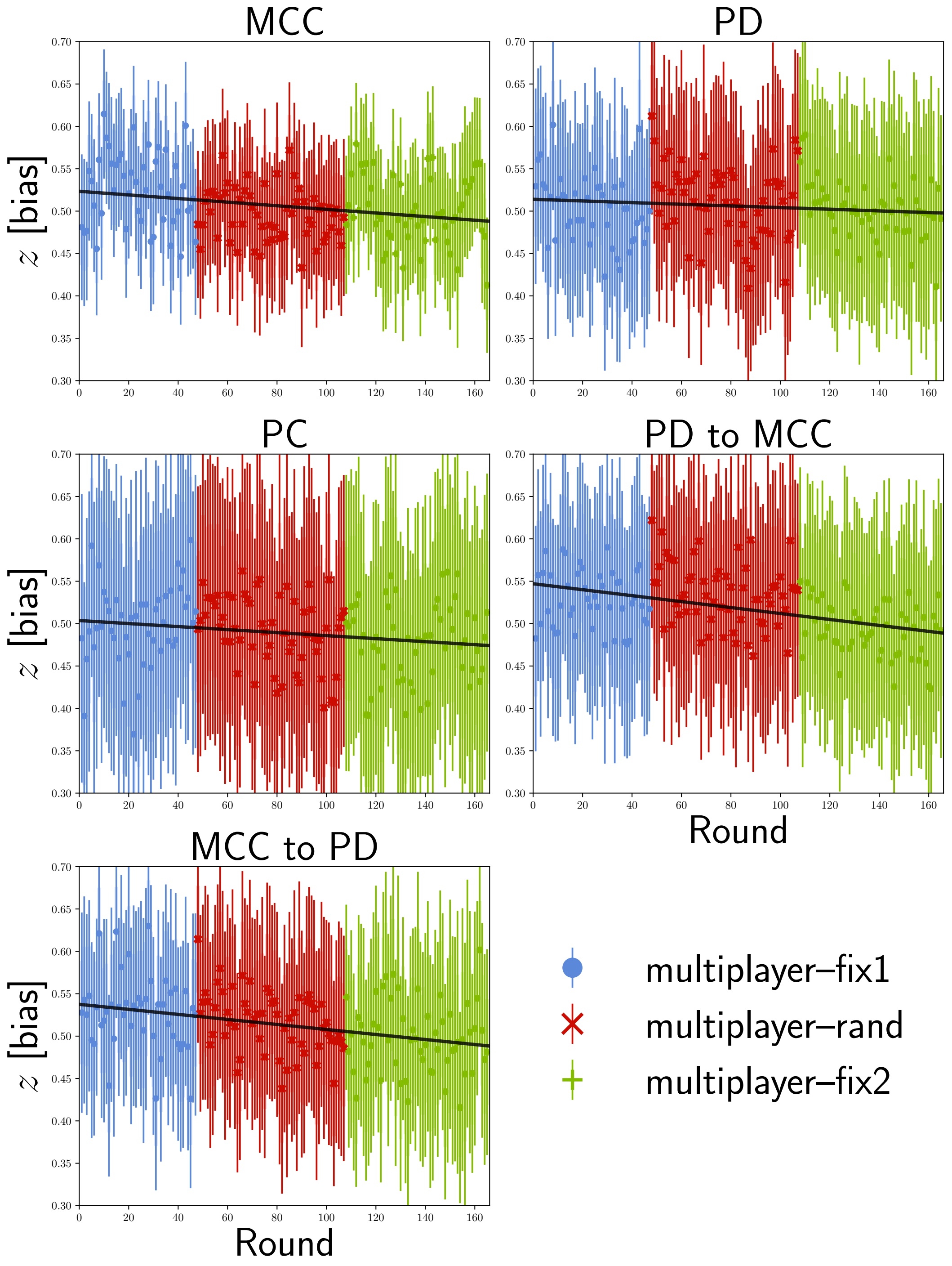

Supplementary Figure 5. The drop in the value of the bias z is independent by the players' strategy. In the multiplayer experiment we can separate players into three types: Moody Conditional Cooperators (MCC), Pure Defectors (PD), and Pure Cooperators (PC) [20]. PD and PC are the players who always defect or cooperate and MCC change their action depending on what their neighbours did in the previous round and what they themselves did in the previous round. Here we separate players by the strategy they picked in fix1 and fix2: in the first three panels we show the results for users who kept the same strategy in both phases, while in the latter two users who switched between two different strategies. In all the cases we observe a similar average drop in the value of the bias z. The progressive emergence of a bias towards defection is therefore not related to the users actions or strategy. Consequently, we argue that it necessarily depends on the experience of playing against a majority of defectors.

	multiplayer fix1	multiplayer rand	multiplayer fix2	pairwise
Rounds	47	60	58	100
$\langle t \rangle$ (sec)	6.73	5.02	4.98	1.59
STD(t) (sec)	4.56	3.55	3.57	2.06
SEM(t) (sec)	0.67	0.46	0.47	0.21
C_R	28.7%	18.5%	22.9%	88.8%
a (sec$^{\frac{1}{2}}$)	5.32 ± 0.03	4.86 ± 0.02	4.73 ± 0.02	3.01 ± 0.04
v (sec$^{-\frac{1}{2}}$)	-0.203 ± 0.005	-0.357 ± 0.006	-0.284 ± 0.006	0.35 ± 0.02
z	0.529 ± 0.004	0.529 ± 0.004	0.509 ± 0.004	0.731 ± 0.006
t_0 (sec)	0.38 ± 0.01	0.340 ± 0.007	0.315 ± 0.005	0.236 ± 0.003
r^2	0.97	0.94	0.95	0.99

Supplementary Table 1. Average and fit values for the whole phases of the multiplayer experiment and for the pairwise experiment These values describe the distribution and fits in Figure 2 and Supplementary Fig. 3. We remark that between the phases fix2 and rand one would not see any significative differences if the study were considering only the moments of the decision times distribution.
